# Supplementary material for: DMRforPairs: identifying Differentially Methylated Regions between unique samples using array based methylation profiles
Source: BMC Bioinformatics. 2014 May 15;15:141. doi: 10.1186/1471-2105-15-141 (PMC4046028; doi:10.1186/1471-2105-15-141)
Supplement: Additional file 2 — DMRforPairs output for the comparison of A431-MCF7 and NA17018-NA17105. Please start from the HTML files in each folder. Available via the BMC Bioinformatics website. [file 1471-2105-15-141-S2.zip › 1394847754114233_MOESM2_ESM/A431_MCF7/figures/16653.pdf]

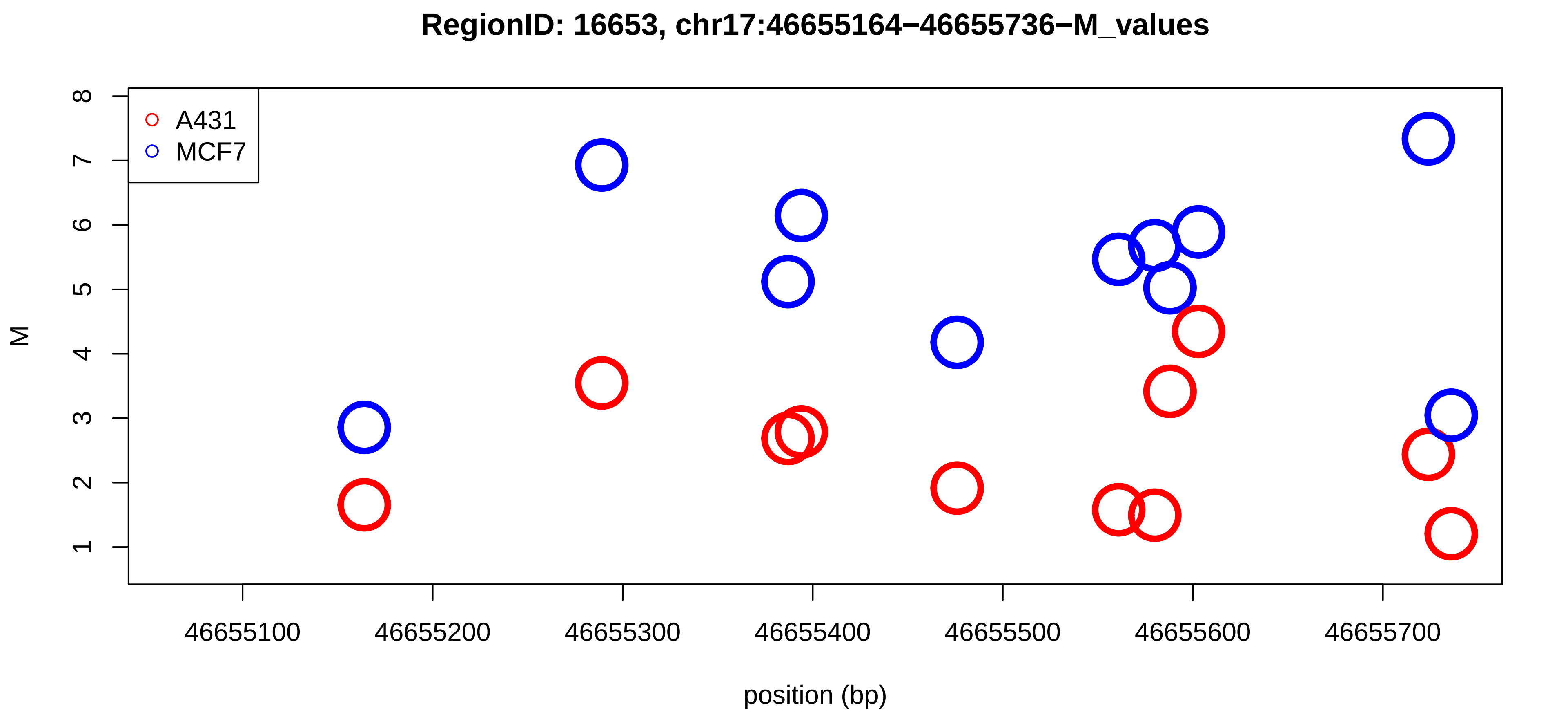
RegionID: 16653, chr17:46655164−46655736−M_values
A431
MCF7
M
1
2
3
4
5
6
7
8
46655100
46655200
46655300
46655400
46655500
46655600
46655700
position (bp)

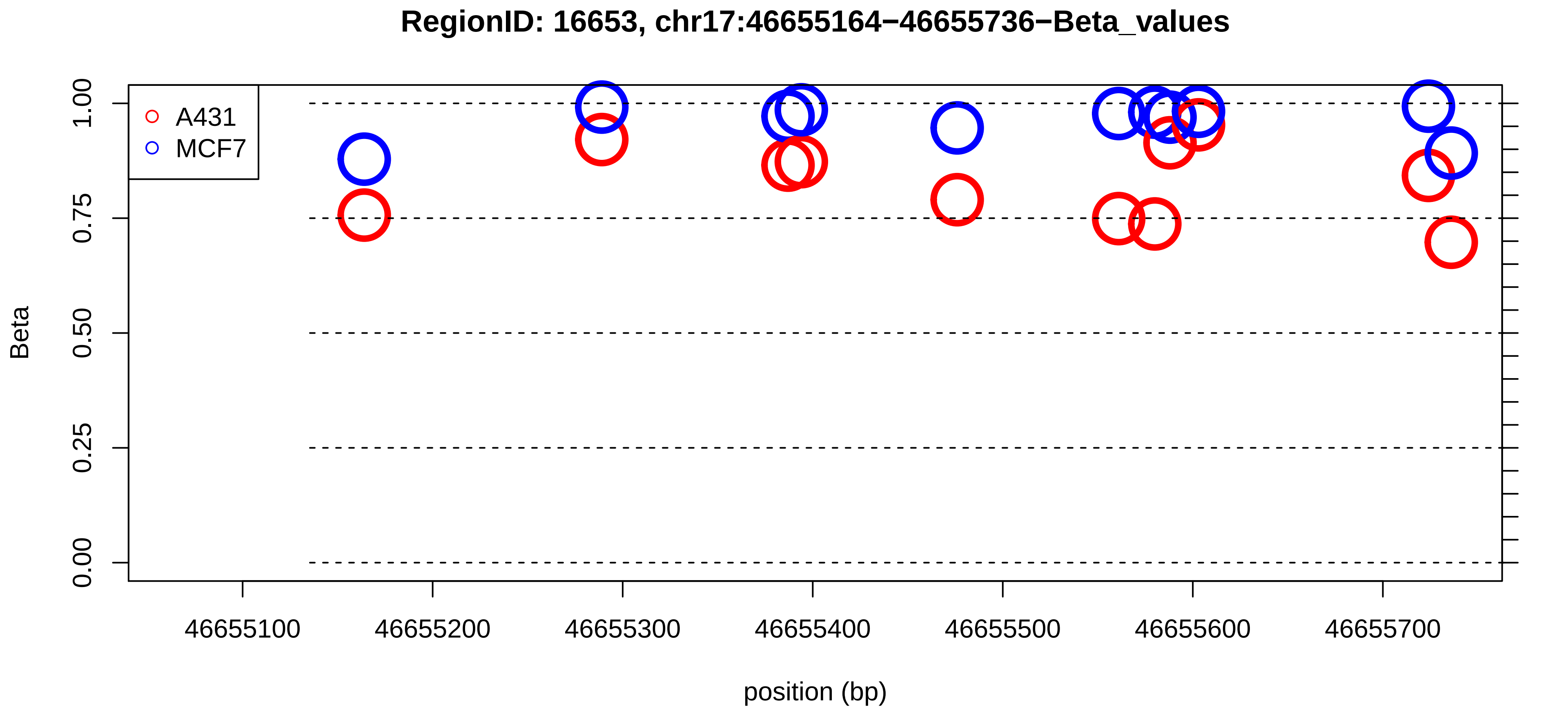
RegionID: 16653, chr17:46655164−46655736−Beta_values
A431
MCF7
Beta
0.00
0.25
0.50
0.75
1.00
46655100
46655200
46655300
46655400
46655500
46655600
46655700
position (bp)

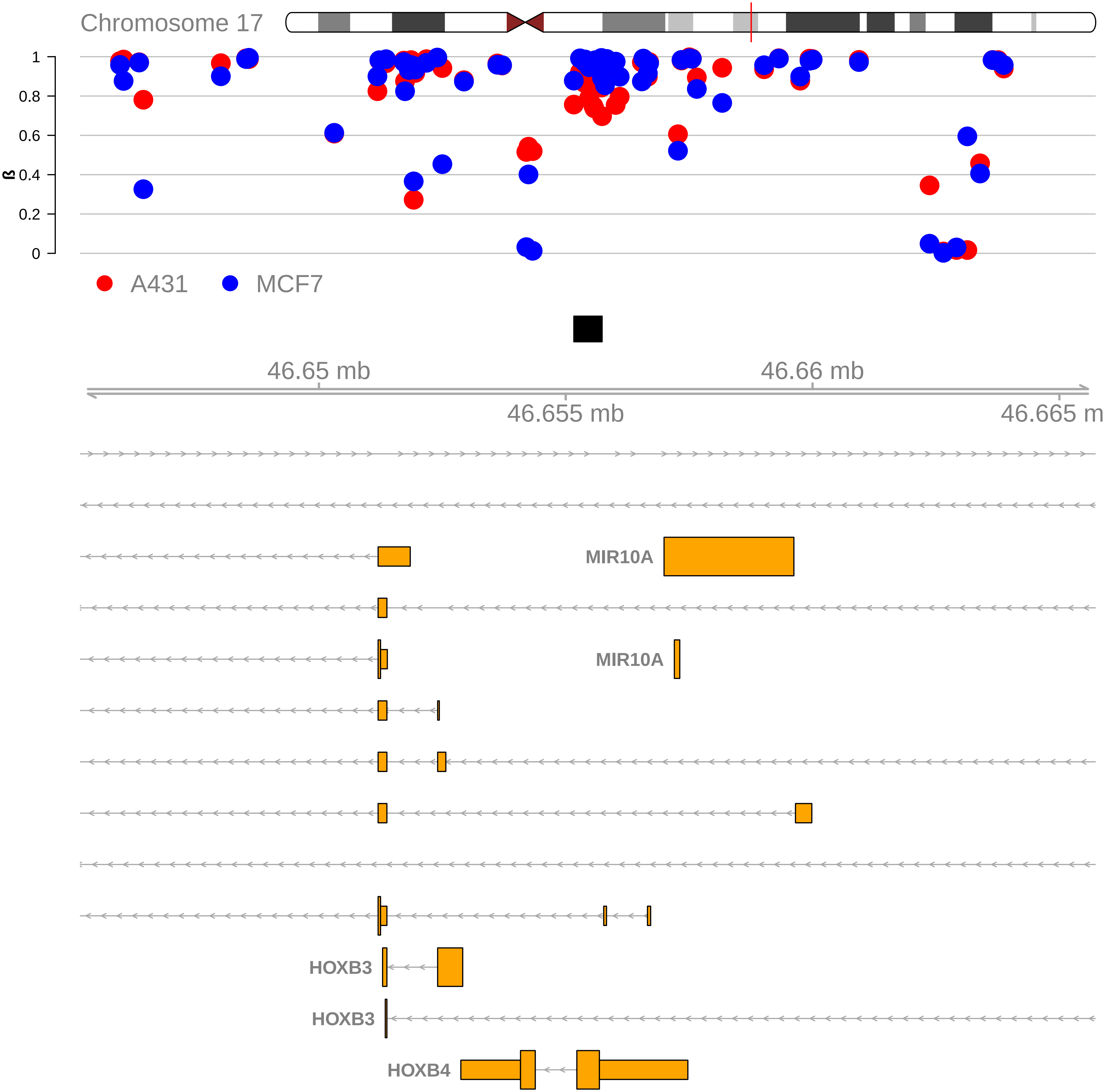

Chromosome 17
1
0.8
0.6
ß 0.4
0.2
0
A431
MCF7
46.65 mb
46.66 mb
46.655 mb
46.665 m
MIR10A
MIR10A
HOXB3
HOXB3
HOXB4
